# The nanotopography of SiO_2_ particles impacts the selectivity and 3D fold of bound allergens

**DOI:** 10.1039/d1nr05958k

**Published:** 2021-12-01

**Authors:** Robert Mills-Goodlet, Litty Johnson, Isabel J. Hoppe, Christof Regl, Mark Geppert, Milena Schenck, Sara Huber, Michael Hauser, Fátima Ferreira, Nicola Hüsing, Christian G. Huber, Hans Brandstetter, Albert Duschl, Martin Himly

**Affiliations:** Dept. Biosciences, Paris Lodron University of Salzburg Austria martin.himly@plus.ac.at; Christian Doppler Laboratory for Innovative Tools for Biosimilar Characterization, Paris Lodron University of Salzburg Austria; Dept. Chemistry and Physics of Materials, Paris Lodron University of Salzburg Austria

## Abstract

A detailed description of the changes that occur during the formation of protein corona represents a fundamental question in nanoscience, given that it not only impacts the behaviour of nanoparticles but also affects the bound proteins. Relevant questions include whether proteins selectively bind particles, whether a specific orientation is preferred for binding, and whether particle binding leads to a modulation of their 3D fold. For allergens, it is important to answer these questions given that all these effects can modify the allergenic response of atopic individuals. These potential impacts on the bound allergen are closely related to the specific properties of the involved nanoparticles. One important property influencing the formation of protein corona is the nanotopography of the particles. Herein, we studied the effect of nanoparticle porosity on allergen binding using mesoporous and non-porous SiO_2_ NPs. We investigated (i) the selectivity of allergen binding from a mixture such as crude pollen extract, (ii) whether allergen binding results in a preferred orientation, (iii) the influence of binding on the conformation of the allergen, and (iv) how the binding affects the allergenic response. Nanotopography was found to play a major role in the formation of protein corona, impacting the physicochemical and biological properties of the NP-bound allergen. The porosity of the surface of the SiO_2_ nanoparticles resulted in a higher binding capacity with pronounced selectivity for (preferentially) binding the major birch pollen allergen Bet v 1. Furthermore, the binding of Bet v 1 to the mesoporous rather than the non-porous SiO_2_ nanoparticles influenced the 3D fold of the protein, resulting in at least partial unfolding. Consequently, this conformational change influenced the allergenic response, as observed by mediator release assays employing the sera of patients and immune effector cells. For an in-depth understanding of the bio-nano interactions, the properties of the particles need to be considered not only regarding the identity and morphology of the material, but also their nanotopography, given that porosity may greatly influence the structure, and hence the biological behaviour of the bound proteins. Thus, thorough structural investigations upon the formation of protein corona are important when considering immunological outcomes, as particle binding can influence the allergenic response elicited by the bound allergen.

## Abstract 2

A detailed description of the changes that occur during the formation of protein corona represents a fundamental question in nanoscience, given that it not only impacts the behaviour of nanoparticles but also affects the bound proteins.

## Graphical abstract

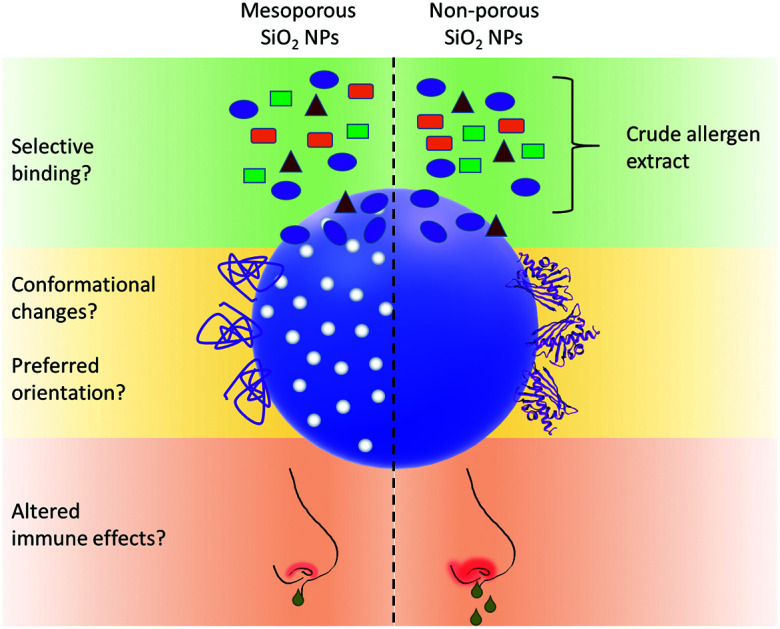

## Introduction

SiO_2_ nanoparticles (NPs) represent the most produced nanoparticles by weight with an estimated production of 1.5 million tons per year.^1^ They are widely used in food additives, cosmetic products, tyres, construction, and agriculture.^2–9^ The high abundance of SiO_2_ NPs in these products can directly increase their presence in the environment, thereby resulting in increased instances of NPs interacting with different entities in the environment. Therefore, there is a higher potential for unintentional human exposure to NPs, either alone or in conjugation with other environmental entities.^10,11^ Proteins, or more specifically, allergens are among the environmental entities that have greater chances to interact with NPs due to their higher abundance in the environment. NPs can efficiently bind allergens to their surface due to their higher free energy levels compared to the bulk material, and thereby form protein corona.^12^ The protein corona greatly influences the biological identity of NPs because upon entering the human body, the first point of contact with biological entities is not the neat NP surface itself, but rather the different proteins, including allergens, forming the corona.^13,14^ Notably, binding to the particle does not only have an impact on the behaviour of the particle, but also on the properties of the attached protein. Accordingly, a number of physicochemical parameters of NPs, such as their size, shape, surface charge, charge density, and chemical functionalisation are involved in the formation of the corona and participate in determining which protein binds more effectively to the NPs and especially in what ratio.^15,16^ The influence of the corona on the biological identity of the NPs makes studying the formation of the corona an important topic in nanoscience.^17,18^

A protein allergen can elicit harmful immune reactions in a limited number of people, which is termed atopics. These people have higher chances of developing allergic symptoms and often display higher total immunoglobulin E (IgE) levels from birth. The past five decades have witnessed an alarming increase in the number of atopics worldwide.^19–21^ Allergic asthma from respiratory allergies constitutes the predominant condition, which affects about 235 million people.^22–24^ These respiratory allergies are caused by airborne allergens, mainly pollen.^25^ Pollen from birch and other members of the *Betulaceae* family represent the major tree pollen in Central and Northern Europe.^26^ The formation of a protein corona, specifically NP-allergen corona, can have a huge impact in the modulation of allergic responses. This can be categorised into different scenarios. In the first scenario, the possible selectivity for a specific component of a crude extract from an allergenic source (*e.g.*, birch pollen) may enrich a specific allergenic entity, consequently inducing a more severe allergic reaction. Secondly, the binding of an allergen to the surface of NPs can modify its 3D structure, thereby altering the epitope recognition by the respective immunoglobulins that mediate recognition by allergic immune effector cells (*i.e.*, mast cells and basophilic granulocytes). In the third scenario, the allergen binds to NPs in a non-randomized binding orientation, leading to either hiding or accumulation of the allergenic epitopes. A fourth scenario could be that the binding of the allergen to NPs results in the formation of neo-epitopes. All these scenarios are relevant for the formulation of novel biologics to circumvent the aggregation and preserve the structural integrity of proteins.^14^ Taken together, the formation of allergen-NP corona can modify the immune response in many ways, either directing a more harmful or protective immune response. Accordingly, previous studies have shown that the binding of allergens to NPs can modify the cellular response against allergen–NP conjugates in various ways.^27–29^

Intrigued by incidental findings when studying mesoporous silica NPs as potential carriers, the aim of this study was to investigate two important aspects in the formation of protein corona, as follows: (i) binding selectivity and (ii) conformational integrity of the biological molecule upon particle binding. The allergen was non-covalently bound to two different model SiO_2_ NPs. The model SiO_2_ NPs utilised differed in their nanotopography, *i.e.*, one was non-porous with a smooth surface (NSNPs) and the other was mesoporous, displaying an uneven interface (MSNPs) for the adsorption of proteins. In particular, in the field of nanofabrication, innovation may be guided or instructed by naturally occurring biomaterials. In this regard, nanotopographic features are an interesting new field of research. Furthermore, cellular and molecular interactions may be regulated these such features.^30^ Birch pollen extract and a highly purified recombinant preparation of the major birch pollen allergen Bet v 1 were coupled with the different SiO_2_ NPs. Our investigations included the elucidation of different binding affinities of the SiO_2_ NPs for different components of the birch pollen extract, given that this may lead to the accumulation of certain allergens on the surface of these particles. Furthermore, following the identification of the major binding protein from the crude extracts, the binding capacities of the model NPs for the specific protein were determined. Moreover, to analyse the influence of particle binding on the 3D fold of proteins, circular dichroism (CD) spectroscopy and a novel enzymatic assay, termed the analytical cascade of enzymes (ACE), were used.^31^ To investigate whether protein-NP binding favours a specific orientation of the bound allergen at the particle surface, limited proteolysis approaches with different proteases were employed. Finally, to uncover modified biological responses such as potential changes in the availability of the allergenic epitopes, direct ELISA and basophil degranulation assays were performed on the particle-bound *vs.* free allergen after incubation with either the allergen–NP conjugates or the unbound allergen.

## Experimental section

### Synthesis and characterisation of SiO_2_ nanoparticles

The non-porous SiO_2_ NPs (NSNPs) were synthesised utilising the Stöber method with tetraethyl orthosilicate (TEOS) *via* an ammonia-catalysed reaction in water and ethanol, as previously described.^27^ For the synthesis of the mesoporous SiO_2_ NPs (MSNPs) a previously described method based on TEOS as the silica source and the incorporation of cetyltrimethylammonium bromide (CTAB) for pore formation was applied.^32^ Briefly, to a mixture of ethanol (2.2 mmol), sodium hydroxide (7 mmol), CTAB (2.7 mmol) and H_2_O, heated to 80 °C, TEOS (20.2 mmol) was added and stirred at 80 °C for 2 h. To determine the Si content in the NPs in an aqueous suspension, the blue silicomolybdic assay adapted for microtiter plates was used, as previously described.^28^ The primary size of NSNPs and MSNPs and the pore size of MSNPs were determined *via* high-resolution transmission electron microscopy (HR-TEM). Briefly, the samples were prepared by pipetting 2 μL of an aqueous suspension (0.01 mg mL^−1^) of the compounds onto lacey carbon-coated Cu TEM grids and air-dried at room temperature. HR-TEM investigation was carried out using a cold-field emission gut JEOL JEM F200 STEM/TEM instrument (JEOL, Freising, Germany) operated at 200 kV. The hydrodynamic diameter of the NPs was determined *via* nanoparticle-tracking analysis using a NanoSight LM10 (Malvern Panalytical, Malvern, United Kingdom), as previously described.^28^ For each sample, 10 videos with a duration of 20 s were captured and analysed. These steps were repeated three times for each sample and the mean value was calculated from three individual measurements. The surface charge of the NPs was measured by diluting the particles to a final concentration of 0.1 mg mL^−1^ in H_2_O and analysing them using a ZetaSizer Nano ZS (Malvern Panalytical, Malvern, United Kingdom). The specific surface area and pore size distributions were calculated *via* nitrogen absorption/desorption measurements. Isotherms were recorded on an ASAP2420 (Micromeritics, Norcross, USA) at 77 K, and prior to the measurements, all samples were degassed at 100 °C for 12 h. The specific surface area was obtained from Brunauer–Emmett–Teller (BET) calculations in the relative pressure range of 0.05–0.3 *p*/*p*_0_. To determine the pore size distribution on the surface of the NPS, the Barrett, Joyner, and Halenda (BJH) method and the absorption branches of the isotherms were used.^33^

### Qualitative and quantitative protein corona determination from crude allergen extract

To determine the potential selectivity of proteins binding to the two types of NPs and their amounts, a procedure comprising three assays was conducted, as follows: (i) sodium dodecyl sulphate-polyacrylamide gel electrophoresis (SDS-PAGE) of both the particle-bound and the unbound protein, followed by densitometric analysis of the resolved protein bands; (ii) spectrophotometric analysis of the particle-bound and unbound protein fractions; and (iii) protein identification by western blotting. For coupling, 500 μg mL^−1^ NPs was incubated with 200 μL birch pollen extract (BPE) (20 mg birch pollen in 5 mL H_2_O) overnight on a rotation wheel. After centrifugation, the proteins from the supernatants and pellets were resolved by SDS-PAGE to determine which fractions of the BPE were bound to the SiO_2_ NPs. For the analysis of the protein binding capacity of the two types of SiO_2_ NPs, Bet v 1 was coupled to the NPs at different concentrations ranging from 10 μg mL^−1^ to 80 μg mL^−1^ (10% to 80% protein per particles (w/w), see Table 1). The mixture was incubated overnight with gentle agitation. The protein–NP conjugates were pelleted *via* centrifugation for 30 min at 16 000*g*. The total protein content of the supernatants (unbound fraction) and the pellets (particle-bound fraction) was determined using the Pierce™ BCA protein assay (Thermo Fischer Scientific, Rockford, USA) according to the manufacturer's instructions. In brief, 25 μL of sample was mixed with 200 μL of working reagent and incubated at 37 °C for 30 min. Thereafter, the absorbance of the samples was measured at 562 nm on an infinite M200 PRO (Tecan, Männedorf, Switzerland).

**Table 1:** Amount of recombinant protein and NPs used in the different experiments

Experiment	Concentration MSNPs	Concentration NSNPs	Concentration Bet v 1	Protein/NP ratio
Binding capacity	100 μg mL^−1^	100 μg mL^−1^	10–80 μg mL^−1^	10–80%
CD spectroscopy	1 mg mL^−1^	1 mg mL^−1^	100 μg mL^−1^	10%
Two-step ACE	4 mg mL^−1^	8 mg mL^−1^	1 mg mL^−1^	12.5–25%
Limited proteolysis MS	400 μg mL^−1^	1 mg mL^−1^	100 μg mL^−1^	10–25%
Bet v 1 ELISA	400 μg mL^−1^	1 mg mL^−1^	100 μg mL^−1^	10.25%
huRBL	0.0004–4000 ng mL^−1^	0.01–10 000 ng mL^−1^	0.0001–1000 ng mL^−1^	10–25%

For identification of the selective NP-binding major component of the birch pollen extract, an anti-Bet v 1 monoclonal antibody, termed BIP 1, was used for immunoblotting. After the NPs and the birch pollen extract incubated overnight and the bound proteins were resolved using SDS-PAGE, the proteins were blotted on a nitrocellulose membrane for 15 min at 15 V *via* a semidry process. Following three washing steps with TBS-T containing 0.1% Tween 20, the blotting membrane was blocked for 1 h at room temperature (RT) in TBS-T containing 5% milk powder (blocking buffer). The blot was washed again and incubated for 1 h at RT with BIP 1 diluted 1 : 5000 in blocking buffer. After an additional washing step, the blot was incubated with anti-mouse IgG and HRP-linked antibody (horse anti-mouse IgG (heavy and light chain) #7076, Cell Signaling, Denver, USA) diluted 1 : 2000 in blocking buffer for 1 h at RT. The blot was washed again and chemiluminescence detection was performed using a SuperSignal™ West Pico PLUS Chemiluminescent Substrate (Thermo Fischer Scientific, Rockford, USA) on a ChemiDoc MP imaging system (Bio Rad, Feldkirchen, Germany).

### Recombinant protein production

The expression and purification of recombinant Bet v 1.0101 were performed following previously described protocols.^27,34,35^

### Protein–nanoparticle coupling procedure

The coupling of protein to the two different types of SiO_2_ NPs was performed by mixing the components and incubating the mixture overnight at 4 °C on a rotational wheel. The specific concentration of the protein and particles varied for the experiments due to their different requirements, which can be found in Table 1.

### Secondary structure determination of NP-bound proteins

Circular dichroism (CD) analysis was performed to determine the structural alterations in the protein upon binding to the two different NP types. The free Bet v 1 and SiO_2_ NP-coupled Bet v 1 samples were analysed in parallel using a JASCO J-815 spectropolarimeter (Jasco, Tokyo, Japan). The CD spectra were recorded at 20 °C in 5 mM sodium phosphate at pH 8. The recorded data was baseline corrected (subtraction of CD measurement with corresponding buffer) and expressed as mean residue molar ellipticity [Θ]_MRW_ for a wavelength range of 190 to 260 nm. The turbidity of the NP samples was negligible. The absorbance readings at all wavelengths were monitored to control the scattering effects by the NPs. To determine the extent of partial protein unfolding due to NP-binding, a sample of 0.5 M urea-stressed Bet v 1, incubated once unbound and once NP-bound, was analysed.

### Enzyme cascade analysis

To determine the structural alterations upon particle binding, the two-step analytical cascade of enzymes (ACE), as previously described by Hollerweger *et al.*,^31^ was performed. This method uses the sequential application of enzymes to amplify the signal of small conformational changes of a protein and makes them detectable using simple laboratory methods. The idea behind this method is that through structural variations, more protease cleavage sites may be accessible for proteolytic cleavage in the first step. In turn, more lysine sidechains are accessible to the second enzyme, microbial transglutaminase, which attaches a biotin-labelled glutamine to the target protein. Consequently, more loosely folded or completely unfolded protein variants will be labelled more strongly. Different minor structural variations or modifications will yield different cleavage patterns. Briefly, the protein–NP conjugates and free proteins were subjected to proteolysis by the protease legumain^36^ (1 : 400 molar ratio of protease to Bet v 1) for 3 h. Afterwards, legumain was inhibited using the specific irreversible inhibitor YVAD-cmk (Bachem, Bubendorf, Switzerland). A biotinylated glutamine donor peptide (150 : 1 molar ratio of peptide to Bet v 1) (Zedira, Darmstadt, Germany) was then covalently crosslinked to the accessible lysine residues using a 1 : 5 molar ratio of microbial transglutaminase (Zedira, Darmstadt, Germany) to Bet v 1 for 2 h. Thereafter, the samples were resolved by SDS-PAGE and blotted on a nitrocellulose membrane. The biotin labelling was analysed using streptavidin poly-HRP employing chemiluminescent detection, as described above. To determine the effect of partial unfolding, Bet v 1 was analysed unstressed and stressed with 0.5 mM urea.

### Limited proteolysis mass spectrometry

To determine the potential preferred orientation of the bound protein, a mass spectrometry (MS)-based limited proteolysis approach was employed. This method determines, if the protease concentration or digestion time represents a limiting factor for the expected enzymatic turn-over, stretches of the protein not fully cleaved into small peptides, and hence protected from enzyme access by NP binding. The free and NP-bound protein (for amounts see Table 1) were subjected to digestion using 0.02 μg mL^−1^ trypsin (Cat# V5111, Promega, Madison, USA) and 0.02 μg mL^−1^ legumain^36^ (produced in house) for varying incubation times ranging from 5 s to 4 h at 37 °C, respectively. Afterwards, the cleaved peptides were isolated from the NPs by centrifugation for 60 min at 16 000*g* and analysed using mass spectrometry coupled to liquid chromatography to determine their sequence. Chromatographic separation of 20 μL sample was carried out on a Thermo Scientific™ UltiMate™ 3000 Rapid Separation system (Thermo Fisher Scientific, Germering, Germany) at a flow rate of 100 μL min^−1^ employing a Thermo Scientific™ Hypersil GOLD™ aQ C18 column (100 × 1.0 mm i.d., 1.9 μm particle size, 175 Å pore size, (Thermo Fisher Scientific, Sunnyvale, CA, USA) operated at a temperature of 50 °C. Mobile phase A was composed of H_2_O + 0.1% formic acid (FA) and mobile phase B of acetonitrile (ACN) + 0.1% FA. The total runtime for one sample was 50 min. The separation started at 1% B for 2 min followed by a linear gradient of 1–5% B in 2 min, 5–10% B in 2 min, 10–35% B in 15 min, 80% B for 4 min, and 1% B for 25 min.

Mass spectrometry was conducted on a Thermo Scientific™ Q Exactive™ Hybrid Quadrupole-Orbitrap™ mass spectrometer equipped with a Thermo Scientific™ Ion Max™ ion source with a heated electrospray ionization (HESI) probe (140 °C), both from Thermo Fisher Scientific (Bremen, Germany). The source heater temperature was set to 140 °C, spray voltage to 3.5 kV, sheath gas flow to 8 arbitrary units, capillary temperature to 300 °C and S-lens RF level to 60.0. Each scan cycle consisted of a full scan in the scan range of *m*/*z* 350–2000 and a resolution setting of 70 000 at *m*/*z* 200, followed by 5 data-dependent higher-energy collisional dissociation (HCD) scans at 29% normalized collision energy at a resolution setting of 17 500 at *m*/*z* 200. For the full scan, the automatic gain control (AGC) target was set to 1 × 10^6^ charges with a maximum injection time of 150 ms, and for the HCD scans the AGC target was 2 × 10^5^ charges with a maximum injection time of 250 ms.

Data acquisition was conducted using Thermo Scientific™ Chromeleon™ 7.2 CDS (Thermo Fisher Scientific, Germering, Germany). For data analysis, MaxQuant 1.6.17.0^37^ was used applying a 1% false discovery rate for peptide identification. The relative quantification of the peptides was based on the intensities of the summed extracted ion current chromatograms of the identified peptides. The sequence coverage map was created with the tool DrawMap from MSTools.^38^

### ELISA

For the determination of the accessibility of the Bet v 1 epitopes when bound to NPs, direct ELISA was performed. Anti-Bet v 1 mAbs BIP 1, 5H8, #2, and #11 were used as the primary detection antibodies.^39,40^ Firstly, a 96-well plate was coated with 10 μg mL^−1^ free or SiO_2_ NP-bound Bet v 1 overnight at 4 °C using 0.2 M bicarbonate buffer at pH 9.5. The plate was then washed three times with PBS containing 0.05% (v/v) Tween20 (wash buffer), and subsequently blocked for 2 h at RT with PBS containing 1% (w/v) BSA (blocking buffer). Afterwards, the plate was washed trice with wash buffer. The primary detection antibody, *i.e.*, mouse IgG, was diluted 1 : 5000 in blocking buffer and incubated for 2 h at RT. Following an additional washing step, the secondary anti-mouse IgG HRP-linked antibody (horse anti-mouse IgG (heavy and light chain) #7076, Cell Signaling, Denver, USA) (diluted 1 : 2000 in blocking buffer) was added and incubated for 2 h at RT. Thereafter, the plate was washed three times with wash buffer and 50 μL of 3,3′,5,5′-tetramethylbenzidine Liquid Substrate (Cat.#: T4444 Sigma-Aldrich, St Louis, USA) was added to each well. After 5–10 min incubation (depending on the colour development), the stop solution (2 M sulphuric acid) was added and the absorbance was measured at 450 nm with a reference wavelength of 570 nm on an infinite M200 PRO plate reader (Tecan, Männedorf, Switzerland).

### Mediator release assay

To evaluate the potential shift in the allergenic potential of the particle-bound *vs.* free Bet v 1, the previously described rat basophil degranulation assay was used.^41^ Briefly, sera from birch pollen-allergic patients were collected for the mediator release assay and selected (*n* = 10) based on their allergen-specific IgE reactivity. The procedure was approved by the local Ethics Committee of the Allergy Clinic Salzburg (No. 415-E/1398/4-2011). The complement system of the sera from the patients was deactivated by incubation with AG-8 cells (ATCC, Germany). Subsequently, human high-affinity IgE receptor (FcεR1) transfected RBL-2H3 (herein referred to as huRBL) cells, were first passively sensitized overnight with the pre-incubated birch pollen-allergic patient serum IgE. The cells were washed with Tyrode's buffer containing 9.5 g L^−1^ Tyrode's salts, 1 g L^−1^ sodium bicarbonate and 0.1% (w/v) BSA. Following washing, the sensitized huRBL cells were incubated with either free or SiO_2_ NP-bound recombinant Bet v 1 for one hour. The protein concentration used for the assay was 0.0001 ng mL^−1^ to 1000 ng mL^−1^ in eight serial dilutions. The release of β-hexosaminidase by degranulation was measured in the supernatant by the addition of the fluorogenic substrate 4-methyl umbelliferyl-*N*-acetyl-*b*-glucosaminide (Sigma, St Louis, USA), which is cleaved by β-hexosaminidase. After 1 h, the reaction was stopped by the addition of 0.2 M glycine buffer pH 10.7. The fluorescence intensity of the cleaved substrate was measured at the excitation wavelength of 360 nm and emission wavelength of 440 nm on an Infinite M200 PRO plate reader (Tecan, Männedorf, Switzerland). The obtained values were expressed as % of the maximum release obtained with 10% Triton X-100-treated cells. To determine the viability of the cells, an MTT assay was performed.

### Statistical evaluation

Data is presented as the mean value and standard deviation (SD) of three individually performed experiments. The statistical analysis between different sample groups was determined by one-way ANOVA followed by a Bonferroni *post-hoc* test, while for two sets of data an unpaired *t*-test was used. Values with *p* < 0.05 were considered statistically significant.

## Results and discussion

### NP properties impact the formation of protein corona – what determines selectivity in protein binding?

**Fig. 1:**
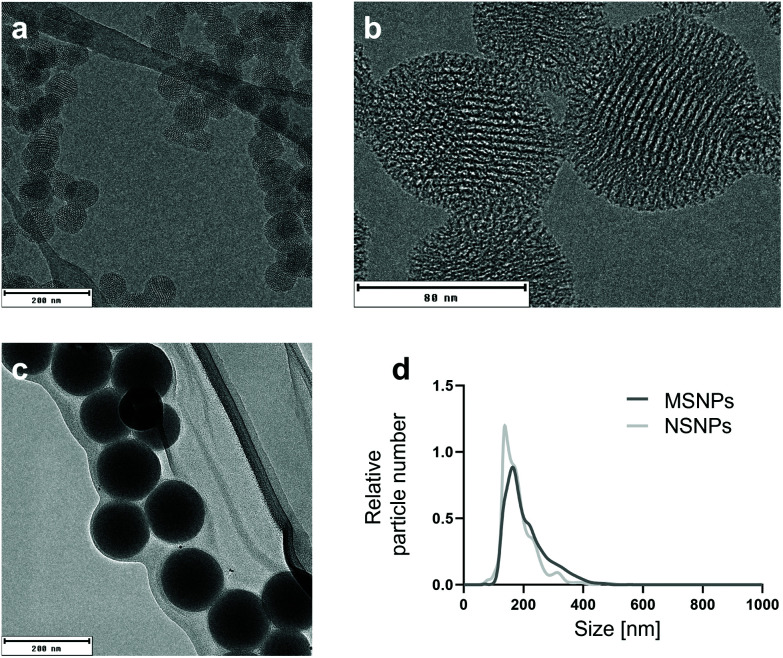
Physicochemical characterisation of the two types of SiO_2_ nanoparticles. (a) Size determination of primary particles of mesoporous SiO_2_ NPs (MSNPs) by transmission electron microscopy (TEM). (b) Close view of MSNPs and pores using high-resolution TEM. (c) Size determination of primary particles of non-porous SiO_2_ NPs (NSNPs) by TEM. (d) Size distribution of both SiO_2_ NPs in solution using nanoparticle tracing analysis (NTA).

To understand the behaviour of NPs, it is necessary to characterise their morphological properties using an array of physicochemical techniques (Table 2), given that these properties can greatly influence how NPs interact with the environment.^42,43^ The particle characterisation *via* nanoparticle tracking analysis demonstrated a narrow size distribution with an average diameter of 201 ± 12 nm for the mesoporous (MSNPs) and 184 ± 41 nm for the non-porous SiO_2_ NPs (NSNPs) (Fig. 1). The zeta potential measurements yielded mean values of −12.7 ± 4.2 mV and −22.1 ± 4.0 mV for MSNPs and NSNPs, respectively. A change in zeta potential was noted after the adsorption of Bet v 1 on the particles, resulting in a value of −25.7 ± 2.4 mV and −25.5 ± 6.3 mV for MSNPs and NSNPs, respectively. The change in zeta potential verified that the protein was effectively (non-covalently) bound to the particles and shielded their surface.^44^ Other groups have previously demonstrated that the formation of a protein corona modifies the net surface charge of the involved NPs, given that the proteins are the major determinant in the surface charge, resulting in negative zeta potential values.^45,46^ A study with gold nanoparticles with different surface charges showed that when NPs are coated with serum proteins, the resulting zeta potential was the same for all the particles.^47^ Surface charge and porosity are both intrinsic morphological properties describing the nanotopography of NPs,^48^ which has previously been shown to greatly influence the cell interactions of nanomaterials.^30^ The surface area and pore size were determined *via* nitrogen sorption using Brunauer–Emmett–Teller analysis, which revealed an area of 1259.8 m^2^ g^−1^ and average pore size of 3.9 nm for MSNPs, while high-resolution TEM revealed a pore size of 4.1 ± 0.3 nm (Fig. 1b). In contrast, NSNPs exhibited a surface area of 24.8 m^2^ g^−1^. All the raw data from the particle characterisation is deposited in the NanoCommons Knowledge Base (https://ssl.biomax.de/nanocommons/cgi/login_bioxm_portal.cgi) under NP01413 (NSNPs) and NP01414 (MSNPs).

**Table 2:** Physicochemical properties of used NPs including particle size, zeta potential without and with bound protein and average pore size

Particle type	Primary size TEM [nm]	Mean size NTA [nm]	Zeta potential [mV]	Zeta potential + Bet v 1 [mV]	Mean pore size BET [nm]	Mean pore size TEM [nm]	Surface area [m^2^ g^−1^]
MSNPs	74.9 ± 9.5	201 ± 12	−12.7 ± 4.2	−25.7 ± 2.4	3.9	4.1 ± 0.3	1259.8
NSNPs	133.8 ± 14.8	184 ± 40	−22.1 ± 4.0	−25.5 ± 6.3	—	—	24.8

**Fig. 2:**
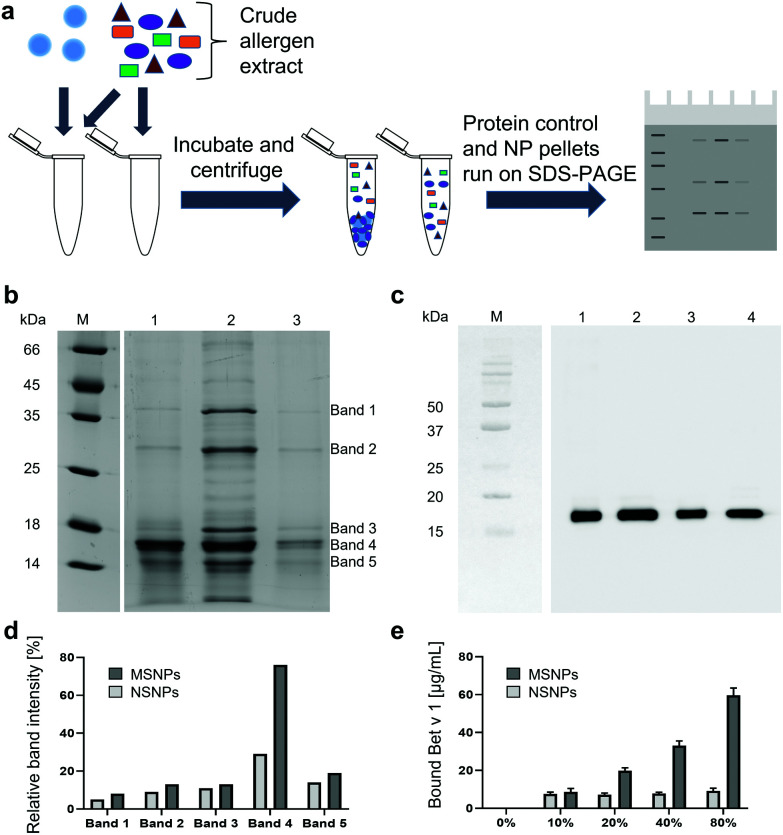
Binding selectivity and Bet v 1-binding capacity of NPs. (a) Schematic representation of the experimental setup. (b) SDS-PAGE of birch pollen extract (BPE) incubated with SiO_2_ NPs. Lane M: protein marker; lane 1: BPE bound to MSNPs; lane 2: BPE only; lane 3: BPE bound to NSNPs. (c) Immunoblot using anti-Bet v 1 monoclonal mouse antibodies for the detection of Bet v 1. Lane M: protein marker; lane 1: BPE bound to MSNPs; lane 2: BPE only; lane 3: BPE bound to NSNPs; and lane 4: recombinant Bet v 1. (d) Intensity of bands 1–5 of BPE bound to SiO_2_ NPs relative to the intensities of BPE only bands. (e) Binding capacity of SiO_2_ NPs for recombinant Bet v 1 (*e.g.*, 10% values refer to 10 μg mL^−1^ protein per 100 μg mL^−1^ NPs).

Following the physicochemical characterisation of the SiO_2_ NPs, their interactions with natural and recombinant allergens were studied. This was conducted by determining the potential selectivity for specific proteins, given that different protein corona compositions can greatly influence the quality of the NP–cell interactions.^49^ Birch pollen extract (BPE) was incubated with the two different SiO_2_ NP suspensions and subjected to detailed binding analyses by comparing the particle-bound protein composition to BPE without SiO_2_ NPs (protein only samples), as depicted in Fig. 2a. From the SDS-PAGE analyses (Fig. 2b), we observed that a protein corresponding to the size of 17 kDa was preferentially bound to the MSNPs (lane 1), and the intensity of this protein band remained almost equal to the BPE compared to all the other bands (lane 2), as depicted in Fig. 2d. Additionally, the intensity of the other prominent BPE bands was markedly reduced in the MSNP corona. However, the comparison of the protein corona of NSNPs (lane 3) did not show any preferential adsorption of protein, but rather exhibited faint bands of the proteins in BPE (lane 2). Subsequently, we compared the relative band intensities of the particle-bound proteins and the proteins of BPE and determined that band 4 exhibited the highest intensity with both SiO_2_ NPs. A relative intensity of 29% was observed for NSNPs, whereas that for MSNPs was 77%. The remaining bands of the proteins bound to the particles showed at least a 2-fold decrease in intensity in NSNPs, and even a 4-fold decrease in MSNPs. Even though both particles selectively bound the protein present in band 4 of BPE, their binding capacity was significantly different. MSNPs bound almost 2-fold more than NSNPs. These results clearly indicate selectivity for particle binding of the 17 kDa protein accounting for band 4, which we identified as the major birch pollen allergen, Bet v 1, employing immunoblotting experiments with the Bet v 1-specific monoclonal antibody BIP 1 (Fig. 2c).^40^ From the blot, it can be concluded that both MSNPs (lane 1) and NSNPs (lane 3) bound Bet v 1, resulting in strong signals at the height of 17 kDa.^50,51^ A signal at the same migration distance was detected in BPE without particles (lane 2) and in the positive control, *i.e.*, recombinant Bet v 1 (lane 4). A similar difference in protein binding behaviour has been previously shown, focusing on similar nanotopography parameters, where MSNPs bound low molecular weight components of foetal bovine serum more efficiently than NSNPs.^52^ It was previously demonstrated that binding selectivity in SiO_2_ NPs was independent of their particle size.^53^ These findings strengthen the hypothesis that the nanotopography parameters studied herein may play a dominant role in the selective binding of protein during the formation of a corona. In the context of interaction with blood plasma proteins, the binding selectivity of NPs has been previously demonstrated, where blood-circulating lipid-based NPs displayed a protein pattern differing from that found in blood plasma without NPs.^54^

### The nanotopography of SiO_2_ NPs determines their binding capacity

To follow-up the structural and biological consequences of these observations in more detail, subsequent experiments were performed using highly purified well-characterised recombinant Bet v 1. The maximum binding capacity of the two different NP types for Bet v 1 was determined and the amount of bound protein was calculated. For NSNPs, an increasing concentration of added protein did not lead to an increase in the maximum protein binding capacity (Fig. 2e). When 100 μg mL^−1^ NSNPs were incubated with either 10, 20, and 40 μg mL^−1^ of Bet v 1, only about 7.6 μg mL^−1^, 7.2 μg mL^−1^ and 7.8 μg mL^−1^ of them were bound, respectively. However, incubation with 80 μg mL^−1^ Bet v 1 led to an increase in the amount of bound protein, *i.e.*, 9.2 μg mL^−1^. The MSNPs behaved differently, where the particles could bind nearly all the offered Bet v 1 for the three lower concentrations (8.7 μg mL^−1^, 19.9 μg mL^−1^, and 33.1 μg mL^−1^ of Bet v 1 bound to the particles with 10, 20 and 40 μg mL^−1^ of protein offered, respectively). The highest concentration, 80 μg mL^−1^ protein, overloaded the particles with only 59.6 μg mL^−1^ being bound (Fig. 2e). Overall, MSNPs showed a higher binding capacity for Bet v 1 compared to NSNPs. This is mainly due to their porosity and the availability of a larger total surface area for protein binding.^55^ Taken together, herein we established that the nanotopography features of NPs can impact their binding capacity for allergens. Considering allergic diseases, safety concerns may arise, given that it has been reported that the allergen load is correlated with the severity of the allergic reaction.^56^ Alternatively, it is well accepted that a reduction of allergic stimuli in the homes of allergic individuals leads to alleviation of allergic symptoms or asthma.^57^ Thus, the increased delivery of Bet v 1 *via* NPs, resulting from selective allergen accumulation on the surface of the particles, can induce a stronger allergic response in sensitive individuals. Moreover, NPs can readily penetrate the epithelial barrier, thus enabling the bound allergen to cross this barrier more easily and enter the body in a larger quantity.^28,58–60^

### Nanotopography impacts the 3D fold of NP-bound allergen

**Fig. 3:**
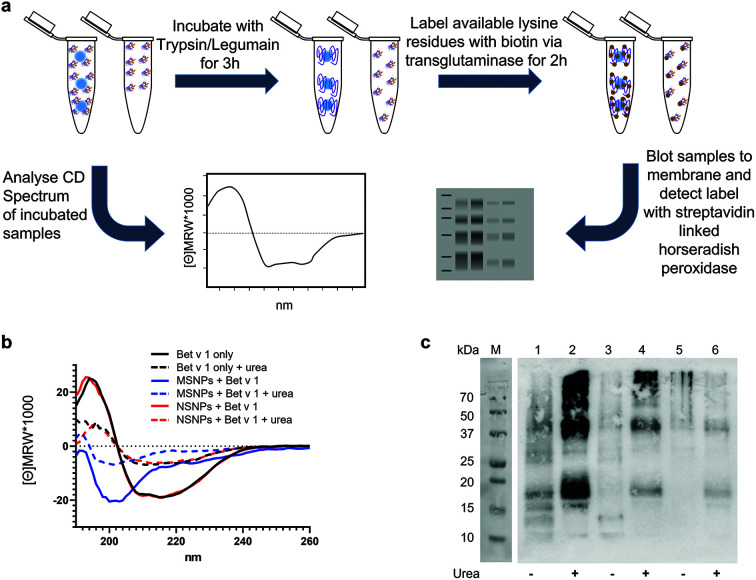
Impact of SiO_2_ NP binding on the 3D fold of the allergen. (a) Schematic representation of the experimental setup. (b) Far UV-circular dichroism spectra of unbound Bet v 1 (Bet v 1 only in black) or bound to MSNPs (blue) and NSNPs (red) spectra were recorded in the presence of 0.5 M urea (dashed lines). (c) Two-step analytical cascade of enzymes assay of unbound Bet v 1 (lanes 5 + 6) or bound to MSNPs (lanes 1 + 2) and NSNPs (lanes 3 + 4), where all samples were analysed untreated (lanes (1, 3, and 5) and upon prior treatment with 0.5 M urea (lanes 2, 4, and 6).

Next, we determined the conformational changes of Bet v 1 following its binding to the two different types of SiO_2_ NPs. Circular dichroism (CD) spectroscopy and a two-step analytical cascade of enzymes (ACE)^31^ were used for the detection of secondary structural changes and their impact on the 3D fold of Bet v 1 (Fig. 3a). The measured CD spectra of Bet v 1 bound to NSNPs were similar to that of the unbound Bet v 1, revealing the same secondary structure contents (alpha helices and beta sheets) (Fig. 3b). However, in Bet v 1 bound to MSNPs, the CD spectra were clearly different from that of the unbound Bet v 1, which displayed a predominant random coil structure with the complete loss of the alpha-helix and beta-sheet structural elements typical for Bet v 1.^61,62^ Subsequently, we determined the degree of unfolding of Bet v 1 in solution *vs*. NP-bound form using CD spectroscopic analysis, where either bound or unbound Bet v 1 was treated with 0.5 M urea to denature the protein (Fig. 3a). The CD spectra of the treated samples exhibited the same trend as that of the untreated samples. Bet v 1 bound to NSNPs gave similar CD spectra as that of the unbound Bet v 1, whereas Bet v 1 bound to MSNPs showed deviations. Nevertheless, in Bet v 1 bound to MSNPs, the CD spectrum of the treated and untreated samples exhibited the slight differences, indicating partial unfolding by MSNPs (Fig. 3b). It was also noted that in all the urea-treated samples, a lower signal intensity was observed, which was probably due to the π interactions of urea with the analyte.^63^ The raw data for the CD analyses can be found under http://doi.org/10.5281/zenodo.4604590.

For further consolidation, the observed structural alterations upon particle binding were additionally investigated using the two-step ACE, which represents a more sensitive measure for partial unfolding than CD spectroscopy. The concept of this method relies on the sequential application of two enzymes with orthogonal functions, resulting in exponential amplification of the signal and indicating conformational changes. The signal can be determined in a simple blot format, which is available in most wet labs without access to CD spectroscopy. When particle-bound and unbound Bet v 1 were subjected to the ACE, a clear difference was evident (Fig. 3c). For the unbound Bet v 1, an ACE signal pattern lacking specific bands was obtained, representing the typical characteristic for a well-folded protein (lane 5). Upon the addition of 0.5 M urea, two distinct bands could be observed on the blot (lane 6) for Bet v 1, and additional signals were detected when the protein was bound to NSNPs (lane 3). For Bet v 1 bound to MSNPs, the highest signal was observed, *i.e.*, higher overall intensity of all bands (lane 1), compared to protein only (lane 5) and the NSNPs-bound Bet v 1 (lane 3) (Fig. 3c). Here, in the ACE experiments, the protein was also stressed by pre-incubation with 0.5 M urea prior to the application of the enzyme cascade. This was carried out to determine if the observed signal intensity could be further increased, in particular for MSNPs. For Bet v 1 bound to MSNPs, the results are consistent with that from CD spectroscopy (Fig. 3b). The ACE signals for the MSNP-bound stressed protein (lane 2) were much more intense compared to the urea-stressed Bet v 1 only (lane 6). For the treated NSNP-bound Bet v 1, the ACE results showed significant differences compared to the treated unbound protein (lane 4 and 6). However, these differences were less intense compared to the MSNP-bound Bet v 1, indicating the more native-like conformation of Bet v 1 when bound to NSNP than to MSNPs. This result is in strong agreement with the results from CD spectroscopy. In addition, it could be detected that all three samples treated with urea (lanes 2, 4, and 6) showed higher ACE signals than the corresponding untreated samples (lanes 1, 3, and 5). In summary, binding to MSNPs showed a significant alteration in the signal compared to the unbound Bet v 1, as revealed by both methods, CD spectroscopy and the ACE assay. For the NSNP-bound Bet v 1, a difference with free Bet v 1 could only be observed using the ACE assay. Thus, we established here that the nanotopography of NPs has an impact on the 3D fold of the tested allergen.

Our findings correlate with previous reports showing the unfolding of proteins upon association with fullerol, as determined by the change in melting temperature and changes in the CD signal.^64^ Particle-induced protein denaturation was also shown for SiO_2_ NPs of varying sizes, where this effect was determined to be size dependent.^65^ The authors demonstrated that particles larger than 150 nm induced conformational changes in proteins, while smaller particles did not, which excluded surface curvature as the main driving force for the observed changes. In our case, this supports the hypothesis that porosity constitutes the main driving force for the observed unfolding and not size, given that both our particles had a relatively similar primary size smaller than 150 nm. Here, it may be speculated that Bet v 1, having a hydrodynamic diameter of approximately 3.8 nm,^66^ may (at least partially) fit into the pores of MSNPs used here, thus impacting both the binding selectivity and increased binding capacity, as discussed above, and leading to partial denaturation. Particle-induced denaturation does not only influence the accessibility of immunologic epitopes on the bound protein, where non-porous SiO_2_ NPs have also been previously shown to decrease the enzymatic activity of lysozyme by inducing conformational changes.^67^ For lysozyme, it was demonstrated that its binding to SiO_2_ NPs leads to the formation of fibrillary aggregates,^68^ which may raise concerns given that the formation of amyloid fibrils has been linked to neuro and non-neurodegenerative diseases.^69^

### Allergen binding to SiO_2_ NPs in randomized orientation is independent of particle porosity

**Fig. 4:**
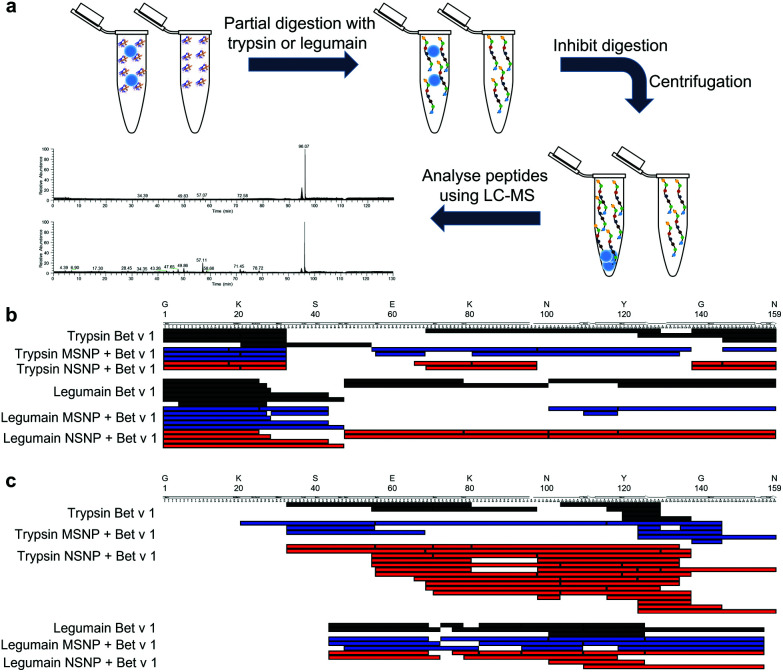
Limited proteolysis of allergen–SiO_2_ NP conjugates *vs.* Bet v 1 only. (a) Schematic representation of the experimental setup. (b) Sequence coverage maps of the ten most intense (by relative abundance) proteolytic peptides upon 5 s partial digestion using trypsin and legumain. (c) Sequence coverage maps of the ten least intense (by relative abundance) proteolytic peptides upon 5 s partial digestion using trypsin and legumain. MSNP–allergen conjugates in red, NSNP conjugates in blue, and unbound Bet v 1 in black.

Next, we determined if the major birch pollen allergen binds to the two tested types of SiO_2_ NPs in a randomized way or rather adopts a preferred orientation. Directed binding is relevant, in particular for allergic individuals, given that this can either accumulate or hide specific allergenic epitopes, eventually critically impacting the allergic response towards the allergen-NP complexes in certain affected individuals.^70^ Therefore, we employed a mass spectrometry (MS)-based limited proteolysis approach using different proteases (Fig. 4a). Conceptually, this method determines specific regions that may be protected from proteolytic digestion due to NP binding. Thus, a significantly lower amount of proteolytic peptides in a specific region indicates a specific binding region and a preferred orientation, given that this region is less accessible to the proteases. The digestion of the samples was carried out using the proteases trypsin and legumain at intervals of 5, 10, 30, 60, 240, 480 s. The entire raw data sets for all time points and both proteases of the MS-based limited proteolysis can be found under http://doi.org/10.5281/zenodo.4604590. From the sequence coverage maps of the most and least intense peptides (Fig. 4b and c, respectively), we could not resolve a single preferred orientation, given that there was no specific region in the amino acid sequence of the bound protein that was protected from the digestion of the proteases. However, this does not necessarily imply that the binding is completely random. A more likely scenario would be that Bet v 1 possesses a small number of preferred orientations displaying similar binding affinities. In a similar study, the reorientation of cytochrome c bound to the surface of SiO_2_ NPs was found to be dependent on the pH.^71^ Nevertheless, the authors could not determine the specific region of the protein involved in binding to the NP surface, but still were able to determine a shift from head-on to side-on binding. Furthermore, a different binding orientation was shown for fibrinogen bound to differently charged gold NPs, as determined by the presence and absence of certain fragments upon SDS-PAGE analysis.^72^ However, both methods described were not applicable in our case, given that Bet v 1 is mostly a globular protein, resulting in too small changes in the corona thickness of different orientations. Hence, the rather small molecular weight of 17 kDa and lack of a single specific protease (contrasting fibrinogen) require a more elaborate analysis that the limited MS-based proteolysis approach.

### Bet v 1 binding to non-porous SiO_2_ NP results in efficient antibody recognition, indicating well-folded Bet v 1 epitopes

**Fig. 5:**
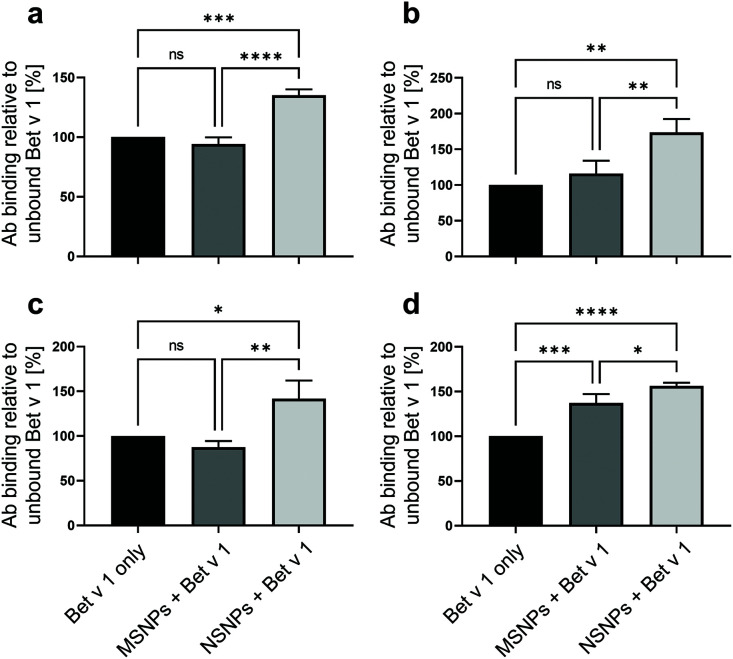
Accessibility of monoclonal antibody (mAb) epitopes of Bet v 1 bound to MSNPs (dark grey bars) and to NSNPs (light grey bars) in comparison to the unbound Bet v 1 control (black bars) determined by ELISA using (a) BIP 1, (b) #2, (c) #11, and (d) 5H8 anti-Bet v 1 mAbs. Values are expressed as % of mAb binding normalized to unbound Bet v 1. Values represent means of *n* = 3 with SD, statistical significance is indicated as ns *p* > 0.05, *p ≤ 0.05, **p ≤ 0.01, and ***p ≤ 0.001, ****p ≤ 0.0001.

It has previously been proposed that NP binding may result in epitope accumulation or hiding when a protein is bound to the surface of nanomaterials.^14^ Therefore, we determined the accessibility of the Bet v 1 epitopes, *i.e.*, antibody recognition sites, by employing direct ELISA using anti-Bet v 1 monoclonal antibodies (mAbs), *i.e.*, BIP 1, 5H8, #2, and #11. These mAbs are known to bind to four different epitopes on the molecule.^39,40^ All samples were normalised for total protein amount bound to the ELISA plate, and thus an observed change in the ELISA readout represented a change in accessibility of the respective epitope, corresponding to the specificity of the used mAb (Fig. 5). For all the tested antibodies, the highest readout was observed for Bet v 1 bound to NSNPs. The ELISA readouts obtained with the different mAbs were 40% to 70% higher for the NSNP-bound samples compared to that for the unbound and MSNP-bound Bet v 1. In the case of MSNPs, a more diverse picture was observed for the different mAbs. Only minor changes in antibody binding were evident for BIP 1 (approx. minus 3%), #11 (approx. minus 15%), and for of #2 (approx. plus 16%), while 5H8 showed a statistically significant increase of approx. plus 37% in antibody binding with respect to the unbound Bet v 1, but still lower than that with NSNPs. Therefore, we concluded that NSNPs potentially induced the local accumulation of the well-folded Bet v 1 epitopes due to an avidity effect, while in MSNPs, the partial denaturation due to the nanotopography counteracted this epitope accumulation effect for two to three out of four different interaction sites on the molecule. It has been previously demonstrated that the accessibility of different antibodies to specific epitopes can be modified by partial unfolding, in the case of food allergens, having an effect on their sensitizing potential.^73^ Similarly, heat denaturation of allergens has been reported to decrease the integrity of IgE epitopes,^74^ evidencing the impact of structural alterations on antibody recognition. Thus, these results confirmed that binding of Bet v 1 to NSNPs resulted in efficient antibody recognition due to its well-folded state at the particle surface, while this state was compromised by the specific nanotopography of MSNPs.

### Biological consequences of protein corona formation – influence on allergenic potential

**Fig. 6:**
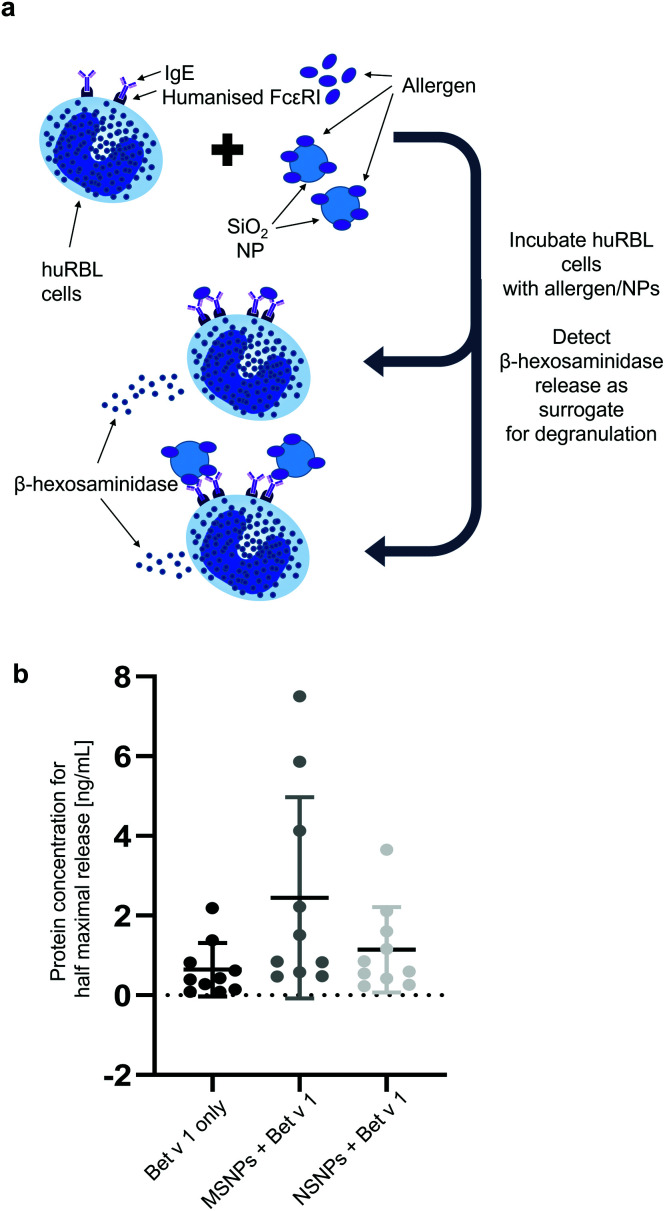
Impact on allergenic response after nanoparticle binding determined by mediator release assay. (a) Schematic representation of the underlying mechanism. (b) Data represented as protein concentrations needed for the half-maximum release of β-hexosaminidase compared to release using 0.1% Triton X-100.

The formation of protein corona can have an impact on the allergic response. Thus, we determined the integrity of the IgE epitopes using a mediator release assay and employing humanized rat basophil leukaemia (huRBL) cells. In this model, the cells were first sensitized with sera from birch pollen-allergic patients, and thereafter incubated with Bet v 1 either bound to SiO_2_ NPs or in its free form. This assay measured the cleavage of the fluorogenic substrate *p*-nitrophenyl-*N*-acetyl-β-glucosaminide by β-hexosaminidase released from the huRBL cells (Fig. 6a). This enzyme is released concomitantly with other mediators, such as histamine, and represents a hallmark of the allergic effector function. The mediator release data is expressed as protein concentration required for achieving the half-maximum release of β-hexosaminidase. We observed that NP binding induced an increase in variability between the sera of different allergic patients. For Bet v 1 bound to the NSNPs, the variability was almost double compared to that of free Bet v 1, and it was 4-fold in the case Bet v 1 bound to MSNPs. The mean concentration for the half-maximum release increased with binding to the NPs. For the unbound Bet v 1, a concentration of only 0.71 ± 0.67 ng mL^−1^, for the NSNP-bound allergen 1.14 ± 1.07 ng mL^−1^ and for the MSNP-bound allergen 2.44 ± 2.53 ng mL^−1^ Bet v 1 were required to achieve the half-maximum release. This highlights a shift in the allergenic potential for the MSNP-bound form. However, these observed trends were dependent on the epitope specificity of the respective allergic donor. One reason for the increased variability is most likely based on the variety of epitopes the patients react to, given that it has been shown that the IgE epitopes, for a number of major allergens, are distributed over the entire allergen surface.^75,76^ The donor-to-donor variability found here was comparable to previous studies, which demonstrated that the patients reacted differently towards gold NP-bound allergens.^29^ Furthermore, as stated above, the different epitopes may be accessible to a varying degree when the protein is bound to the particles either due to certain preferred orientations or due to blocking by neighbouring molecules when they are tightly packed on the particle surface or by entering the pores. By binding to MSNPs, an additional factor comes into play, namely the observed unfolding of Bet v 1, which seemed to further modulate or destroy certain epitopes, leading to an increase in variability and decrease in allergenic response.^77^ This hypothesis was confirmed by our observation that Bet v 1 was less allergenic when bound to MSNPs. The impact of conformational changes on the allergenicity has already been shown for ovalbumin, where conformational changes after heating and glycation reduced its potential allergenicity.^78^ Our finding that allergen binding to SiO_2_ NPs impacts the allergenic behaviour is also consistent with previous findings, which reported that the binding of Bet v 1 to SiO_2_ NPs modulates the cellular response against this major birch pollen allergen, where SiO_2_ NPs association leads to an increase in pro-inflammatory cytokine expression.^28^ Furthermore, SiO_2_ NPs were shown to exert an immunosuppressive effect in a mouse model of allergic contact dermatitis by decreasing the 2,4-dinitrofluorobenzene (DNFB)-induced cytokine expression and epidermal hyperplasia.^79^

## Conclusions

In this study, we compared the influence of the nanotopography of two different types of SiO_2_ NPs, namely mesoporous NPs (MSNP) *vs.* NPs possessing a smooth surface (NSNPs), on the formation of protein corona by applying allergens as a model system. Selectivity for binding the major birch pollen allergen Bet v 1 was demonstrated. Additionally, we showed that MSNPs, not NSNPs, led to at least partial unfolding of the protein upon non-covalent conjugation to the particles, as evidenced by CD spectroscopy and the two-step ACE assay. A single preferred orientation for allergen binding could not be identified using the limited MS-based proteolysis approach. Nevertheless, the nanotopography of the particles had an impact on the availability of specific epitopes, which we addressed by ELISA with four Bet v 1 specific mAbs with different epitope specificities. This observation was further confirmed by a highly allergen-specific assay, *i.e.*, degranulation of allergic effector cells primed with sera from human allergic patients. This modification in the 3D structure and the biological and immunological properties of the Bet v 1 due to the nanotopography of the SiO_2_ NPs stresses to a further degree that all the physicochemical properties of NPs need to be reported as comprehensively as possible when investigating the impact of protein–NP interactions. Particles of similar core chemistry possessing a different nanotopography, such as porosity and surface charge, can lead to quite different biological outcomes. Moreover, we proved that protein corona investigations based on model systems employing allergens display the capacity to determine the bio-nano interactions in versatile ways. Finally, the alterations in the allergenic potential upon particle binding may have wide-ranging effects for affected individuals, and therefore are also of major interest for nanosafety considerations.

## Ethics approval and consent to participate

The collection and usage of patient samples for the huRBL assay was approved by the local Ethics Committee of the Allergy Clinic Salzburg (No. 415-E/1398/4-2011).

## Data availability

All data generated or analysed within this study is included in this published article. Physico-chemical characterisation data including relevant metadata for the MSNPs (NP01414) and NSNPs (NP01413) has been deposited in the NanoCommons Knowledge Base (https://ssl.biomax.de/nanocommons/cgi/login_bioxm_portal.cgi) and raw data for the MS-based limited proteolysis approach is accessible at Zenodo (http://doi.org/10.5281/zenodo.4604590).

## Author contributions

Most of the experimental work was performed by RMG, who also prepared the first draft of the manuscript and figures. LJ was involved in the experimental work and analysis of data for Fig. 6. IJH and HB provided tools and expertise for the two-step ACE assay (Fig. 3b). MG carried out TEM, DLS, and zeta potential measurements (Fig. 1). MS and NH provided SiO_2_ NPs and performed BET measurements. CR conducted the mass spectrometry and CH provided the suitable facilities and the limited proteolysis concept. SH, MiHa, and FF provided tools and expertise for Fig. 6. MaHi conceived the study. MaHi and AD supervised the work. AD provided infrastructure and funding for the study. All authors were involved in reviewing and editing of the manuscript.

## Conflicts of interest

The authors declare the following competing financial interest(s): HB holds a patent for the ACE method (https://patents.google.com/patent/US10775384B2/en?oq=US10775384B2).

## Supplementary Material
